# Unscreened: Urgent and Emergent Surgical Outcomes in the Early COVID-19 Pandemic

**DOI:** 10.7759/cureus.11878

**Published:** 2020-12-03

**Authors:** Christina Colosimo, Jason Kelly, James Coker, Sidra Bhuller, Eric Ballman, Christina Baker-Sparr, James Yon, Brendon Cornett, Oliwier Dziadkowiec, John Weaver

**Affiliations:** 1 General Surgery, HCA HealthONE - Sky Ridge Medical Center, Lone Tree, USA; 2 Administration, HCA HealthONE - Sky Ridge Medical Center, Lone Tree, USA; 3 Clinical Data Analytics, HCA HealthONE - Sky Ridge Medical Center, Lone Tree, USA; 4 General Surgery, HCA HealthONE – Swedish Medical Center, Englewood, USA; 5 Graduate Medical Education, HCA Healthcare, Lone Tree, USA

**Keywords:** covid-19, sars-cov-2 pandemic, testing, urgent surgeries, emergent surgeries

## Abstract

Background

Creating useful recommendations for changes in surgical protocols during the severe acute respiratory syndrome coronavirus 2 (SARS-CoV-2) pandemic has been difficult due to a lack of studies based on representative samples. This study evaluates the clinical outcomes and characteristics of patients undergoing urgent or emergent surgeries.

Methods

This is a multi-center (eight-hospital), retrospective, observational study of urgent and emergent surgical patients from Colorado and Kansas, the United States, in the early stages of the SARS-CoV-2 pandemic. Patient groups were divided based on their coronavirus disease 2019 (COVID-19) status: positive, negative and untested. COVID-19 testing was performed after the surgery if patients were symptomatic.

Results

The analysis includes 5,547 patients who underwent surgery from March 1, 2020 to May 17, 2020. Seventy-four percent (4,096) were not tested for COVID-19 due to lack of symptoms. Out of the 1,451 tested patients, 1,412 tested negative, and 39 tested positive. Out of all the patients who tested positive, 69.23% were admitted to the intensive care unit (ICU), whereas 16.72% of untested and 21.25% of the negative patients. The invasive ventilation rate for the patients that tested positive was 46.15%, 4.22% for untested, and 8.85% for patients who tested negative. The mortality rate in the positive group was 7.69%, 1.10% in the untested group, and 1.56% in the positive group.

Conclusion

Patients who tested positive for COVID-19 had worse clinical outcomes than patients who tested negative and untested. We recommend creating criteria for testing based on patient characteristics and surgical procedure rather than testing all patients awaiting surgery; this would allow us to conserve resources moving forward.

## Introduction

Clinical decision making during the severe acute respiratory syndrome coronavirus 2 (SARS-CoV-2; the causative virus of coronavirus disease 2019 [COVID-19]) global pandemic has been extremely challenging due to the possible transmission of the virus through asymptomatic carriers and lack of data regarding post-surgical clinical outcomes for COVID-19 positive patients [1, 2]. Studies published during the pandemic created a proliferation of anecdotal evidence regarding outcomes of COVID-19 positive patients [2, 3]. These smaller, underpowered studies have led to broad clinical recommendations and unproven changes in surgical protocols. Performing surgery on asymptomatic patients who have not been tested for COVID-19 with no outcomes data is one prominent example [4].

The American Society of Anesthesiologists’ statement on perioperative testing notes that “Patients who are infected with SARS-CoV-2, the virus responsible for the COVID-19 disease, have higher perioperative morbidity and mortality” [5]. The long incubation period for the SARS-CoV-2 virus has raised concerns that patients may undergo surgery during this infected but asymptomatic phase [6, 7]. Therefore, universal testing efforts and protocols have been created to help alleviate concerns of patient and surgical staff safety [6, 8].

Some of the current recommendations are based on three small retrospective studies [4, 9, 10]. Aminian et al. reported a 50% mortality rate in four COVID-19 positive patients who underwent urgent surgery in Iran. Two of the four patients were readmitted with COVID-19 approximately two weeks postoperatively, raising the possibility that they may not have been infected at the time of surgery. The third patient was scheduled for surgery but became symptomatic prior to surgery and was never in an operating room [9].

Lei et al. identified 34 operative patients with confirmed COVID-19, of which 15 (44.1%) required intensive care unit (ICU) care and had an overall mortality rate of 20.5% [4]. The authors compared the mortality rate of the 34 surgical patients to the overall mortality rate of COVID-19 positive patients. This was comparing a population with potentially less disease burden to surgical patients. The paper also does not describe the mortality rate for non-surgical COVID-19 patients in their facility. Thus, the paper was not able to compare the mortality rates of surgical and non-surgical patients, making it impossible to claim a higher mortality rate in COVID-19 positive patients who underwent surgery. The study also suggested that surgery accelerates the symptoms of COVID-19 by examining median time from surgery to symptoms but does not take into account that patients had been admitted to the hospital for a median of two-and-a-half days prior to surgery. Including those extra two-and-a-half days, the median time to first symptoms suggests that patients were infected on the day of admission and had the same median five days from infection to onset of symptoms as reported elsewhere in China. Since we do not know when the patients were infected, we do not know if the symptoms were accelerated, and they could have been infected in the hospital on day one. 

The only study that examined patient outcomes from patients who tested positive for COVID-19 and underwent surgery that was available at the time this manuscript was written came from the COVIDSurg Collaborative on May 29, 2020. The authors reviewed data from 1,128 patients from 235 hospitals in 24 countries that underwent emergency or elective surgeries between January 1, 2020 and March 31, 2020. The authors found the 30-day mortality rate to be 23.8%, with pulmonary complications occurring in 51.2% [11]. The patient demographics affecting the mortality rate in the paper included male gender and age 70 years or older. The COVIDSurg Collaborative study also did not include a comparison of unscreened patients to patients testing negative and surgical outcomes. The paper does not provide a representative emergency or elective surgery sample from each of the 235 hospitals.

As elective surgical procedures resume in healthcare systems in the United States and worldwide, more high-quality information is needed to address both patient and medical provider concerns. This information would also help redefine the guidelines and recommendations for patient care in the COVID-19 era.

Our study evaluates 5,547 patients undergoing urgent or emergent surgeries in eight hospitals in Denver, Colorado, and Wichita, Kansas, which are part of a large healthcare system. Outcomes of this study have the potential to generate insights on resuming elective surgical cases and to quantify the benefit of perioperative testing.

## Materials and methods

Study design and participants

This is a multi-center, retrospective, observational study within one healthcare system in Colorado (six hospitals total) and Kansas (two hospitals total), located in the United States in the early stages of the SARS-CoV-2 pandemic. The study examined 5,547 patients who underwent surgery from March 1, 2020 to May 17, 2020. All patients that underwent surgery in the eight facilities were included in the study. During this time, all eight hospitals had the same policy to put a hold on elective surgery and only allow urgent and emergent cases. All patient data was de-identified by the quality and patient safety data manager before analysis. IRB exemption was obtained for the study.

The patient population consisted of urgent or emergent surgical patients in one regional healthcare system. Patient groups were divided based on their COVID-19 status: positive, negative, and untested. Data was collected from a de-identified Electronic Health Record (EHR) during the early stages of the SARS-CoV-2 (COVID-19) pandemic in Colorado and Kansas in the United States. COVID-19 testing was not performed prior to surgery, and all symptoms or complications related to the infection became apparent postoperatively.

Statistical analysis

Due to large differences in sample sizes, particularly with the smaller COVID-19 positive group, Cramér’s V was used to utilize effect size in addition to the p-value as our measure of statistical differences between groups following the Chi-Square test. Cramér’s V describes the magnitude of the difference, rather than just whether the likelihood of the difference being observed was by chance (p-value). Effect size cut-off values (Table 1) for less than small, small and medium used Cohen’s recommendation with two degrees of freedom [12].

**Table 1:** Effect size cut-off values

Degree of freedom	Small	Medium	Large
1	0.10	0.30	0.50
2	0.07	0.21	0.35

Kruskal-Wallis rank test was used to compare medians among the groups for continuous variables because they were not normally distributed. These normality violations are entirely consistent with variables such as length of stay (LOS) and physiological characteristics. As with the Chi-Squared test, we utilized effect size (Eta-squared) to describe the between-group differences rather than p-value.

Holm-Bonferroni p-value adjustment method was used to penalize/adjust the p-value for multiple comparisons [13]. Additionally, alpha level was set to 0.01 in order to further protect from falsely rejecting the null-hypothesis with a large sample size.

Outcome measures

Three groups (untested, COVID-19 positive, and COVID-19 negative patients) were compared based on their demographic characteristics and primary surgeries, bedside procedures, ICU usage, comorbidities, medication use, symptoms, discharge and LOS, and complications.

## Results

During the first COVID-19 peak between March 1, 2020 and May 17, 2020, 5,547 patients underwent urgent or emergent procedures in a total of eight hospitals located in Denver, Colorado, and Wichita, Kansas. Of those patients, 1,451 (26%) were tested for COVID-19, and 39 (2.7%) had positive test results.

No meaningful differences in median age were noted between the three groups (Table 2). More females were found in the untested (54.5%) and negative (52.4%) groups, and more males in the positive group (61.5%). All of the patients’ operations were filed under the surgical subspecialty of their primary surgery. Listed below are the percentages of each group who underwent surgery in that surgical subspecialty. The majority of the patients had either general (33.99%) or orthopedic surgery (22.34%). We defined general surgery as general acute care surgery (appendectomies, cholecystectomies, herniorrhaphy, etc.) and colorectal (colon and anorectal surgeries). There were significant differences between all three groups regarding the percentage of general surgeries (p<0.01), with 53.85% of the positive patients undergoing a general surgical procedure. A significantly higher proportion of patients in the negative group underwent neurosurgery (p< 0.01). Ophthalmology recorded only four surgeries, of which all patients were in the untested group. Although there are statistically significant percentage differences between the three groups, the magnitude of these differences can be described as “small”.

**Table 2:** Demographic characteristics and primary surgeries ‡: Eta-Squared (η2) was used to compare the Kruskal-Wallis one-way analysis of variance. ENT - ear, nose and throat; OBGYN - obstetrics and gynecology; OMFS - oral and maxillofacial surgery; IQR - interquartile range

	Untested (n=4096)	Negative (n=1412)	Positive (n=39)	P-values	Cramér’s V	Effect size
Age				1.00	0.001^‡^	< Small
Median (IQR)	60 (33-87)	61 (38-84)	62 (37-87)			
Sex				1.00	0.023	< Small
Female	2233 (54.53%)	740 (52.41%)	15 (38.46%)			
Male	1862 (45.47%)	672 (47.59%)	24 (61.54%)			
Primary surgery						
Cardio-thoracic	106 (2.59%	54 (3.82%)	0 (0%)	1.00	0.035	< Small
Colorectal	261 (6.37%)	73 (5.17%)	2 (5.13%)	1.00	0.022	< Small
Endocrine	31 (0.76%)	8 (0.57%)	0 (0%)	1.00	0.012	< Small
Endoscopy	236 (5.76%)	61 (4.32%)	2 (5.13%)	1.00	0.028	< Small
ENT	23 (0.56%)	15 (1.06%)	0 (0%)	1.00	0.027	< Small
General	1332 (32.52%)	366 (25.92%)	21 (53.85%)	<0.01	0.075	= Small
Neurosurgery	548 (13.38%)	283 (20.04%)	1 (2.56%)	<0.01	0.086	= Small
OBGYN	237 (5.45%)	73 (5.45%)	2 (5.13%)	1.00	0.007	< Small
OMFS	19 (0.46%)	4 (0.28%)	1 (2.56%)	1.00	0.030	< Small
Ophthalmology	4 (0.10%)	0 (0%)	0 (0%)			
Orthopedic	885 (21.61%)	348 (24.65%)	6 (15.38%)	1.00	0.035	< Small
Plastic	51 (1.25%)	24 (1.70%)	1 (2.56%)	1.00	0.019	< Small
Transplants	6 (0.15%)	9 (0.64%)	0 (0%)	0.86	0.041	< Small
Urology	285 (6.96%)	72 (5.10%)	1 (2.56%)	1.00	0.036	< Small
Vascular	72 (1.76%)	18 (1.27%)	2 (5.13%)	1.00	0.028	< Small

Table 3 shows a between-group comparison of all recorded bedside procedures. Overall, the frequency of bedside procedures was low in this sample of patients. The most common procedure performed for all groups was central line placement. The positive group had a significantly higher proportion of central line placements than the other two groups (p<0.001), but this difference (effect size) is classified as “small”.

**Table 3:** Bedside procedures

	Untested (n=4096)	Negative (n=1412)	Positive (n=39)	P-values	Cramér’s V	Effect size
Central line	119 (2.91%)	54 (3.82%)	9 (23.08%)	<0.01	0.096	= Small
Chest tube placement	36 (0.88%)	13 (0.92%)	0 (0%)	1.00	0.008	< Small
Midline	1 (0.02%)	3 (0.21%)	0 (0%)	1.00	0.031	< Small
Pacemaker	1 (0.02%)	2 (0.14%)	0 (0%)	1.00	0.022	< Small
Placement of intracardiac defibrillator	1 (0.02%)	0 (0%)	0 (0%)	··	··	··
Placement of intracardiac pacemaker	6 (0.15%)	1 (0.07%)	1 (2.56%)	1.00	0.054	< Small
Pleural drainage endoscopy	26 (0.63%)	12 (0.85%)	1 (2.56%)	1.00	0.022	< Small
Thoracotomy	5 (0.12%)	2 (0.14%)	0 (0%)	1.00	0.004	< Small
Tracheostomy	16 (0.39%)	10 (0.71%)	2 (5.13%)	0.40	0.058	< Small

Of the three groups, the majority of the positive patients went to the ICU (69.23%) with an average LOS of 18 days (Table 4), whereas 16.72% of the untested group and 21.25% of the negative group were admitted to the ICU. The LOS for the untested and negative groups was 2.62 and 2.94 days, respectively. The positive group differed significantly from the negative and untested group when comparing ICU admissions (p<0.001) and ICU LOS (p<0.001). There was no significant difference between the negative and untested groups when it came to ICU admissions (p=0.625) and ICU LOS (p=0.328). Non-invasive ventilation was defined as requiring the use of bilevel positive airway pressure (BiPAP) or continuous positive airway pressure (CPAP), while invasive ventilation was defined as endotracheal intubation. Overall, a higher percentage of patients who tested positive for COVID-19 required ventilators (Table 4). Forty-six percent of the patients in the positive group required invasive ventilation compared to 4.22% of patients in the untested group (p<0.001), and 8.85% in the negative group (p<0.001) (Table 4). One patient required extracorporeal membrane oxygenation (ECMO) during their hospital admission in the negative group. The difference between the untested and negative groups was not statistically significant (p=0.152). There were also statistically significant differences between all three groups when it came to the use of noninvasive ventilators (p<0.01). According to the effect size measure, the between-group differences in magnitude can be described as “small”. The median days on invasive ventilation for the positive group was 17.71 compared to 2.08 in the negative group and 2.10 in the untested group.

**Table 4:** Intensive care unit usage ‡: Eta-Squared (η2) was used to compare the Kruskal-Wallis one-way analysis of variance. ICU - intensive care unit; IQR - interquartile range

	Untested (n=4096)	Negative (n=1412)	Positive (n=39)	P-values	Cramér’s V	Effect size
Admitted to ICU	685 (16.72%)	300 (21.25%)	27 (69.23%)	<0.01	0.122	= Small
ICU length of stay, days - median (IQR)	2.62 (0-6.79)	2.94 (0-8.53)	18.21 (0-46.14)	<0.01	0.005^‡^	< Small
Invasive ventilation	173 (4.22%)	125 (8.85%)	18 (46.15%)	<0.01	0.171	= Small
Noninvasive ventilation	117 (2.86%)	68 (4.82%)	7 (17.95%)	<0.01	0.081	= Small
Days on noninvasive ventilator – median (IQR)	3.21 (0-12.83)	2.99 (0-9.58)	5.75 (0-32.42)	1.00	0.009^‡^	< Small
Days on invasive ventilator – median (IQR)	2.08 (0-6.27)	2.10 (0-8.37)	17.71 (0-53.10)	0.02	0.044^‡^	< Small
Extracorporeal membrane oxygenation	0 (0%)	1 (0.23%)	0 (0%)			

The most common comorbidities associated with the positive group listed in descending order were a history of smoking, anemia, diabetes, and hypertension (Table 5). In both the untested and negative groups, the two most common comorbidities were a history of smoking and hypertension (Table 5). There was a significantly higher proportion of patients diagnosed with anemia in the positive group than in the negative (p<0.001) and untested (p<0.001) groups. There was no significant difference between the negative and untested groups (p=0.163). These differences were “small” in magnitude (V=0.075).

**Table 5:** Comorbidities

	Untested (n=4096)	Negative (n=1412)	Positive (n=39)	P-values	Cramér’s V	Effect size
Any comorbidity	1440 (35.16%)	500 (35.41%)	20 (51.28%)	1.00	0.028	< Small
Alcohol abuse	43 (1.05%)	20 (1.42%)	0 (0%)	1.00	0.018	< Small
Anemia	180 (4.40%)	75 (5.31%)	9 (23.08%)	<0.01	0.075	= Small
Cancer	42 (1.03%)	12 (0.85%)	1 (2.56%)	1.00	0.015	< Small
Cardiac disease	37 (0.90%)	23 (1.63%)	1 (2.56%)	1.00	0.033	< Small
Chronic pulmonary disease	162 (3.96%)	72 (5.10%)	6 (15.38%)	0.17	0.052	< Small
Congestive heart failure	128 (3.13%)	48 (3.40%)	5 (12.82%)	1.00	0.046	< Small
Depression	155 (3.78%)	62 (4.39%)	3 (7.69%)	1.00	0.021	< Small
Diabetes	238 (5.81%)	95 (6.73%)	8 (20.51%)	0.16	0.053	< Small
Hypertension	534 (13.04%)	227 (16.08%)	8 (20.51%)	0.48	0.042	< Small
Hypothyroidism	162 (4.46%)	63 (4.46%)	1 (2.56%)	1.00	0.013	< Small
Liver disease	30 (0.78%)	7 (1.63%)	2 (5.13%)	0.76	0.044	< Small
Malignancy	32 (0.78%)	7 (0.50%)	2 (5.13%)	1.00	0.046	< Small
Obesity	166 (4.05%)	78 (5.52%)	4 (10.26%)	0.80	0.039	< Small
Peripheral vascular disease	85 (2.08%)	31 (2.20%)	3 (7.69%)	1.00	0.032	< Small
Renal failure	142 (3.47%)	60 (4.25%)	4 (10.26%)	1.00	0.034	< Small
Rheumatoid arthritis	53 (1.29%)	16 (1.13%)	0 (0%)	1.00	0.011	< Small
Any smoking	851 (20.78%)	256 (18.13%)	10 (25.64%)	1.00	0.031	< Small

When examining the pharmaceutical treatments of patients infected with COVID-19, antibiotics were used the most in all three groups. Patients who tested positive for COVID-19 required a higher proportion of antibiotics than patients who tested negative (p<0.001) and the untested patients (p<0.001). Additionally, more patients who tested negative required antibiotics than patients who were not tested (p<0.01). A higher percentage of patients in the positive group than in the negative (p=0.01) and untested (p<0.001) received antivirals (Table 6). There was also a significant difference in glucocorticoids between the untested and negative group (p<0.001). These differences were “small“ in magnitude.

**Table 6:** Medication use

	Untested (n=4096)	Negative (n=1412)	Positive (n=39)	P-values	Cramér’s V	Effect size
Antibiotics	1319 (32.21%)	512 (36.26%)	32 (82.05%)	0.008	0.094	= Small
Antivirals	117 (2.86%)	70 (4.96%)	5 (12.82%)	0.008	0.066	= Small
Glucocorticoids	867 (21.17%)	384 (27.20%)	15 (38.46%)	0.008	0.070	= Small
Immunoglobulin	28 (0.68%)	22 (1.56%)	0 (0%)	0.008	0.041	< Small

The most common symptoms in the positive group were fever (33.33%), cough (16.38%), and shortness of breath (12.82%). In the untested group, symptoms included fever (9.18%), chills (3.88%), and cough (3.78%) (Table 7). In the negative group, results showed fever (10.34%), cough (7.37%), and chills (3.54%). A higher percentage of patients in the positive group had a fever than in both negative (p<0.001) and untested (p<0.001) groups. There was no significant difference between negative and untested groups. There was a significant difference in cough occurrence between all three groups (p<0.01). There was no significant difference in the presence of cough as a symptom between negative and positive patients (p=0.113). There were significant differences between all three groups in the presence of “shortness of breath” (p<0.01), though the difference between the negative and positive groups was barely significant (p<0.01). These differences were “small“ in magnitude.

**Table 7:** Symptoms

	Untested (n=4096)	Negative (n=1412)	Positive (n=39)	P-values	Cramér’s V	Effect size
Abdominal pain	65 (1.59%)	15 (1.06%)	0 (0%)	1.00	0.022	< Small
Acute respiratory failure	2 (0.05%)	0 (0%)	0 (0%)			
Chest pain	51 (1.25%)	17 (1.20%)	0 (0%)	1.00	0.010	< Small
Chills	159 (3.88%)	50 (3.54%)	0 (0%)	1.00	0.018	< Small
Cough	155 (3.78%)	104 (7.37%)	6 (16.38%)	<0.01	0.084	= Small
Diarrhea	27 (0.66%)	10 (0.71%)	0 (0%)	1.00	0.007	< Small
Dyspnea	47 (1.15%)	22 (1.56%)	2 (5.13%)	1.00	0.033	< Small
Emesis	118 (2.88%)	37 (2.62%)	0 (0%)	1.00	0.016	< Small
Fatigue	15 (0.37%)	3 (0.21%)	2 (5.13%)	0.51	0.068	< Small
Fever	376 (9.18%)	146 (10.34%)	13 (33.33%)	<0.01	0.070	< Small
Malaise	1 (0.02%)	1 (0.07%)	0 (0%)	1.00	0.011	< Small
Myalgia	1 (0.02%)	2 (0.14%)	0 (0%)	1.00	0.022	< Small
Other pain	53 (1.29%)	10 (0.71%)	1 (2.56%)	1.00	0.026	< Small
Palpitations	16 (0.39%)	6 (0.42%)	0 (0%)	1.00	0.006	< Small
Shortness of breath	80 (1.95%)	47 (3.33%)	5 (12.82%)	<0.01	0.070	< Small

One-hundred percent of the patients in the untested group, 94.12% of the negative group, and 79.49% of the positive group were discharged from the hospital (Table 8). These differences are statistically significant (p<0.01). At the time of this study, some patients remain hospitalized. The mortality rate in the positive group was 7.69% compared to only 1.10% in the untested group and 1.56% in the positive group, though these differences were not statistically significant. The LOS was found to be higher in the positive population with a median LOS of 15.42 days compared to 2.38 days in the untested group and 2.46 days in the negative group, but these differences were not statistically significant. When comparing 30-day readmission rates, the positive group had the highest at 10.26%, the untested group was 7.76%, and the negative group was 6.73%; these differences were statistically significant (p<0.01). The discharge disposition is listed below in Table 8. The most common discharge was to home with 70.07% in the untested population, 63.39% in the negative group, and 30.77% in the positive group. Home discharge proportions differed significantly between all three groups (p<0.001). All the differences were “small” in magnitude (Table 8).

**Table 8:** Discharge and length of stay ‡: Eta-Squared (η2) was used to compare the Kruskal-Wallis one-way analysis of variance. IQR - interquartile range

	Untested (n=4096)	Negative (n=1412)	Positive (n=39)	P-values	Cramér’s V	Effect size
Discharged	4069 (99.34%)	1329 (94.12%)	31 (79.49%)	<0.01	0.190	= Small
Expired	45 (1.10%)	22 (1.56%)	3 (7.69%)	0.52	0.052	< Small
Total length of stay, days – median (IQR)	2.38 (0-5.96)	2.46 (0-6.79)	15.42 (0-32.80)	1.00	0.010^‡^	= Small
Readmitted	318 (7.76%)	95 (6.73%)	4 (10.26%)	<0.01	0.192	= Small
Discharge disposition						
Left against advice	26 (0.63%)	7 (0.50%)	1 (2.56%)	1.00	0.022	< Small
Expired	46 (1.12%)	22 (1.56%)	3 (7.69%)	0.52	0.051	< Small
Home	2870 (70.07%)	895 (63.39%)	12 (30.77%)	<0.01	0.092	= Small
Home with home health service	477 (11.65%)	163 (11.54%)	3 (7.69%)	1.00	0.010	< Small
Hospice	45 (1.10%)	18 (1.27%)	3 (7.69%)	1.00	0.051	< Small
Other hospital	12 (0.29%)	2 (0.14%)	0 (0%)	1.00	0.014	< Small
Remanded to law enforcement	6 (0.15%)	4 (0.28%)	0 (0%)	1.00	0.015	< Small
Long term care facility	38 (0.93%)	22 (1.56%)	2 (5.13%)	0.99	0.041	< Small
Skilled nursing facility	381 (9.30%)	112 (7.93%)	4 (10.26%)	1.00	0.021	< Small
Psychiatric facility	6 (0.15%)	2 (0.14%)	0 (0%)	1.00	0.003	< Small
Rehabilitation	160 (3.91%)	73 (5.17%)	2 (5.13%)	1.00	0.028	< Small
Unknown	29 (0.71%)	92 (6.52%)	9 (23.08%)	<0.01	0.203	= Small

Acute kidney failure and cardiac complications were the most frequently reported complications among all three groups (Table 9). The only statistically significant difference between groups was in the proportion of patients with pulmonary complications (p<0.001), with positive patients having the highest proportion. Effect size assessment revealed no differences in magnitude between the three groups that can be classified as greater than “small”.

**Table 9:** Complications SIRS - systemic inflammatory response syndrome

	Untested (n=4096)	Negative (n=1412)	Positive (n=39)	P-values	Cramér’s V	Effect size
Acute kidney failure	416 (10.16%)	168 (11.9%)	12 (30.77%)	0.02	0.060	< Small
Cardiac complications	377 (9.2%)	142 (10.06%)	4 (10.26%)	1.00	0.013	< Small
Infection	1 (0.02%)	2 (0.14%)	0 (0%)	1.00	0.022	< Small
Pulmonary complications	48 (1.17%)	39 (2.76%)	5 (12.82%)	<0.01	0.091	= Small
SIRS and septic shock	127 (3.1%)	56 (3.97%)	6 (15.38%)	0.08	0.059	< Small
Urinary incontinence	31 (0.76%)	14 (0.99%)	0 (0%)	1.00	0.014	< Small
Urinary injury	11 (0.27%)	1 (0.07%)	0 (0%)	1.00	0.019	< Small
Urinary tract infection	94 (2.29%)	43 (3.05%)	3 (7.69%)	1.00	0.035	< Small

## Discussion

To our knowledge, this is the first paper to investigate both the tested and untested patient populations of all urgent and emergent surgeries performed in a regional healthcare system during the COVID-19 pandemic. All currently available data suggests that SARS-CoV-2 infection can lead to prolonged ICU care, increased ventilator requirements, and a higher mortality rate following surgery [4, 14, 15]. The aim of this study was to examine 5,547 patients from eight hospitals who underwent urgent and emergent surgeries from March 1, 2020 to May 17, 2020. The patients were divided into three groups: untested for COVID-19, COVID-19 positive, and COVID-19 negative patients. Their clinical outcomes and characteristics were compared.

In our study, which is the largest published cohort of its kind to date, the untested population was found to have similar outcomes to those who tested negative for COVID-19. At the time this data was collected, all patients were not routinely tested for the virus. Testing was performed on symptomatic patients (febrile, cough, respiratory distress, etc.) postoperatively since these patients came in with urgent or emergent issues requiring immediate procedures. 

It is important to note that we decided to present our findings using both effect sizes (Cramer’s V) and adjusted p-values because it describes the magnitude and probability of the between-group differences more appropriately, especially when comparing groups with such large sample size differences. Methodological flaws related to the utilization of unadjusted p-values with large sample sizes have been widely covered in other publications [16].

One-hundred percent of the untested population was discharged from the hospital compared to 94.1% of negative patients and 79.5% of the positive group. At the time of this manuscript, some patients were still admitted; thus, outcomes could not be reached on all of the cohort. This fact may skew some of the results, such as the ventilator time and LOS, since there may be patients that are still being ventilated in the ICU with the potential for further complications; however, the majority of patients have a record of a clinical outcome making this a good representation of the cohorts.

When comparing this paper to other contemporary studies, the mortality rate was 7.69% in the COVID-19 positive patients. This is a lower rate than what was reported by COVIDSurg Collaborative 23.8%, Lei and colleagues 20.5%, and 50% mentioned by Aminian and colleagues [4, 9, 11]. The LOS was found to be similar between the COVIDSurg Collaborative and our study. The LOS in our study was 15.42 days, and the COVIDSurg Collaborative study reported a LOS of 16 days in their emergency surgery group. It is important to note that the COVIDSurge Collaborative had missing invasive ventilator data on 916 out of 1,115 patients. In our study, rather than dividing the duration of ventilation into categorical groups, with “≥72” as the longest category, we tracked the actual number of days on an invasive ventilator; thus, it is difficult to compare our study to the COVIDSurge Collaborative directly. The median number of days on invasive ventilator support for COVID-19 positive patients in our study was 17.71 days. Additionally, as opposed to 18.2% of patients in our study, 34.3% of patients in studies of COVIDSurg Collaborative and 44.1% of patients in Lei et al. were admitted to the ICU.

A study by Zhou and colleagues investigated risk factors associated with increased mortality from COVID-19. Older age was found to have an odds ratio of 1.10, with a median age of 69 for in-hospital mortality and comorbidities such as hypertension and diabetes [17]. Wu and colleagues found that patients with comorbidities such as hypertension and diabetes were more likely to develop acute respiratory distress syndrome (ARDS) and older patients were more likely to progress from ARDS to death (hazard ratio 3.26) [18]. They classified older age as 65 or older. The most common comorbidities associated with our COVID-19 positive group listed in descending order were a history of smoking, anemia, diabetes, and hypertension. Our study had diabetes and hypertension in common with the above studies, whereas none of the studies evaluated for a past medical history of anemia. Only Wu et al. examined smoking history in their study, but the rate was much lower - 6% versus 25.64% in our positive group [18]. 

A systematic review of 43 clinical symptoms showed that the most common symptoms were fever (83.3%), cough (60.3%), and fatigue (38.0%) [19]. The most common symptoms of the COVID-19 positive group in our study were fever (33.33%), cough (16.38%), and shortness of breath (12.82%). Our study also identified fever and cough as the most prevalent symptoms for the positive group, but they were in lower percentages.

Lei et al. complication rates included ARDS (32.4%), shock (29.4%), secondary infection (29.4%), arrhythmia (23.5%), acute cardiac injury (14.7%), and acute kidney injury (5.9%) [4]. The COVIDSurg Collaborative study found that 51.2% of patients had pulmonary complications [11]. Our positive group’s most common complications were acute kidney injury (30.77%), systemic inflammatory response syndrome (SIRS), septic shock (15.38%), and pulmonary complications (12.82%). Therefore, our pulmonary complications were significantly lower than the COVIDSurg Collaborative study.

General and orthopedic surgeries were the most common primary surgeries performed. This is similar to what was reported in the COVIDSurg Collaborative study, with their top two primary surgeries being gastrointestinal and general surgery and orthopedics [11].

As hospitals began to reopen for elective surgery in the Denver and Wichita areas, new protocols were created. Currently, all patients are required to have a preoperative COVID-19 test performed. After being tested, all patients are to self-quarantine prior to the planned procedure. During that time, a daily temperature log must be kept. Only the patients who have a negative test are cleared for surgery. As stated above, the patients included in this study were only tested if they were symptomatic postoperatively. There is a possibility that some patients in the untested population had COVID-19 but were asymptomatic. In a recent study of 78 patients who tested positive for COVID-19 in Wuhan, China, 33 (42%) were asymptomatic [20]. Additionally, recent studies also suggest that the sensitivity and specificity of reverse transcription-polymerase chain reaction (RT-PCR) tests improve when patients actually start experiencing symptoms and are likely worse in the asymptomatic individuals or in the asymptomatic disease stage [21, 22]. Therefore, since the untested group had the least amount of admissions to the ICU, less time on the ventilator, and all the patients were discharged from the hospital, it raises the question: do we need to test everyone?

New COVID-19 protocols require many resources to test patients: staff to collect the samples, labs to run the samples, and staff to monitor results. Surgeries may even be canceled for COVID-19 positive asymptomatic patients. These recommendations add to the financial and social burden to both the patient and the hospital where the procedure is to be performed. This group of hospitals spends $11,000 a day on tests. That doesn't include the cost in terms of OR protocols, personal protective equipment (PPE), and staff safety. In our study, the untested group had better outcomes than the negative group. Therefore, the question remains if the expense and the resource utilization is worth it if the presumptive asymptomatic COVID-19 positive patients do not have negative clinical results. Therefore, we recommend stratifying perioperative risk for patients and improving the cost in terms of perioperative testing moving forward with elective surgery. 

## Conclusions

We propose, as an alternative, to create criteria to select asymptomatic patients for testing. Patients with comorbidities that have been shown to increase mortality rate (hypertension, diabetes, etc.) would be tested preoperatively. In addition, we could also rationalize testing patients that are older than 65 since they have an increased risk, as discussed above. Testing could also be stratified based on the type of surgical procedure performed, pending better outcomes data on individual surgeries. This approach would be another alternative to help save scarce resources instead of testing every patient preoperatively going forward. We understand as a precaution testing every patient or treating patients as a presumed positive is important at this time to prevent spread. Our goal is to start discussing a plan for going forward, and we understand that this plan won't be put into full effect until we have either heard immunity or a vaccine in place to protect staff and patients from the asymptomatic spread. We believe our study opens the door to further studies that look at outcomes of asymptomatic COVID-19 patients who test positive postoperatively, and we plan to investigate these outcomes in the future. 
